# *Amamiclytus wuxingensis* sp. nov. (Coleoptera, Cerambycidae), the third species of the genus from mainland China

**DOI:** 10.3897/zookeys.889.38909

**Published:** 2019-11-14

**Authors:** Shulin Yang, Cha Wang

**Affiliations:** 1 School of Life Sciences, Guizhou Normal University, Guiyang, Guizhou 550001, China Guizhou Normal University Guiyang China

**Keywords:** longhorn beetle, Cerambycinae, Clytini, taxonomy

## Abstract

*Amamiclytus wuxingensis***sp. nov.**, the third species of the genus from mainland China, is described and illustrated with specimens collected from Wuxing Village, Leishan County, Guizhou Province, China. Distribution and grouping of the new species are discussed and the key to the East Asian species of the genus is updated to accommodate species recorded from mainland China.

## Introduction

*Amamiclytus* Ohbayashi, 1964 (Cerambycidae, Cerambycinae, Clytini) is a genus of small-bodied longhorn beetles with black, glossy bodies and white pubescent maculations on the elytra (Niisato and Han 2011). Species of this genus can be distinguished from species of similar genera, e.g., *Raphuma* Pascoe, 1858 and *Demonax* Thomson, 1861, by their widely separated antennal cavities, dilated apical segments of maxillary palpi in the male, hairy hind tibiae and unique structures on the male genitalia, i.e., minute or medium-sized spinous spicules behind crescent-like sclerites on the base of the endophallus, and dense minute serrate or crenulate spicules on the apical part of the endophallus but without a pair of sclerotized lines formed by spinous spicules on the apical part of endophallus, as in species of the genus *Demonax* (Niisato and Han 2011, 2013). There are 19 taxa described from Asia, including India, Sri Lanka, China, Thailand, Laos, Vietnam and Ryukyu Islands (Tavakilian and Chevillotte 2018). Species of this genus were firstly reported for mainland China in 2013 with *Amamiclytus wenshuani* Niisato & Han, 2013 and *Amamiclytus limaticollis* (Gressitt, 1939) (Niisato and Han 2013). To date, there are eight species recorded for China, including two from the mainland and six from Taiwan (Niisato and Han 2013; Tavakilian and Chevillotte 2018). The ninth species of the genus for China, which is the third species for mainland China and the first for Guizhou Province, is described hereafter with specimens collected from Wuxing Village, Leishan County, Guizhou Province.

## Materials and methods

Specimens were collected by net sweeping on a flowering bird cherry (*Prunus* sp.). Collected specimens were glued onto pinned paper cards. Labels were handwritten in Chinese. All materials, including the holotype, are preserved in the School of Life Sciences, Guizhou Normal University, Guiyang, China (**GZNULS**).

An AmScope SM-4TZ stereomicroscope was used for specimen observation and dissection. Photos for the adult habitus were taken using a Canon EOS 6D digital camera with a Canon MP-E 65 mm lens. Male genitalia were photographed with an Olympus DP22 under an Olympus SZX7 stereomicroscope.

Abbreviations for maculations consisting of white pubescence follow Niisato and Han (2011) and are listed for reference here: **Pb**: basal band on pronotum; **B**: basal bands near elytral bases; **S**: sutural spot on elytra behind scutellum; **La** : lateral bands before middle of elytra; **Lp**: transverse bands behind middle of elytra; **A**: apical bands of elytra; **Msl**: lateral maculation of mesosternum; **Mss**: maculation on mesosternal process; **Mta**: L-shaped band along apical margin of metathorax; **V1–V4**: lateral bands along apical margins of abdominal ventrites 1–4.

## Taxonomy

### Amamiclytus wuxingensis sp. nov.

Taxon classificationAnimaliaColeopteraCerambycidae

8BC6BD2F-5CD3-5C40-B83D-EC81D97EB1A8

http://zoobank.org/A63B961D-D1E6-42D9-AB48-61979EB5801E

Figs 1 , 2

#### Type locality.

Wuxing Village, Leishan County, Guizhou Province, China. 26°21.36'N, 108°01.82'E, altitude ca 1190 m.

#### Type series.

***Holotype*** male, glued on paper card, with genitalia in a separate microvial. Original label: “中国贵州省雷山县五星村/蔷薇科稠李属植物/东经：108°01.82'，北纬：26°21.36' / 2019年4月6日/杨书林采” [Wuxing Village, Leishan County, Guizhou Province, China / flowering bird cherry (*Prunus* sp.) / 26°21.36'N, 108°01.82'E. / 6 April 2019 / Shulin Yang leg.] (GZNULS). HOLOTYPE / Amamiclytus / wuxingensis / Shulin Yang [red handwritten label]. ***Paratype*** 2 males, same data as holotype.

#### Differential diagnosis.

*Amamiclytus wuxingensis* sp. nov. should be grouped into Group III proposed by Niisato and Han (2011) along with *Amamiclytus juni* Niisato & Han, 2011, *Amamiclytus yulongi* Niisato & Han, 2011 and *A. limaticollis*. They share common body and genitalia characters; short, broadened and matted body, rather transverse pronotum with distinctly arcuate sides, without white pubescence near the basal margin, median struts about half the length of median lobe, and parameres about 2/5 the length of tegmen.

*Amamiclytus wuxingensis* sp. nov. can be distinguished from most of its congeners, including *A. yulongi* and *A. limaticollis* of the same group, by the La extending forward and reaching to S. The La does not extend toward and reach S in most of the other congeners.

*Amamiclytus juni* and *Amamiclytus mimicus* Holzschuh, 2018 also have La extending towards and reaching S as in *A. wuxingensis* sp. nov. *A. wuxingensis* sp. nov. differs from *A. juni* in the a) La is distinctly rounded arcuate while less rounded and obliquely turning from the middle of the elytron towards S in *A. juni*; b) Lp transverse while slightly arcuate in *A. juni*; c) Ps absent while present in *A. juni*; d) Mss sparse while completely absent in *A. juni*. Furthermore, characters of male genitalia, e.g., the widened part at the base of the parameres and the pointed apex of the dorsal plate of the median lobe, can be used to differentiate *A. wuxingensis* sp. nov. from *A. juni*. Tergite eight is as wide as sternite eight and its apex is concave at the middle in *A. wuxingensis* sp. nov. Tergite eight is much wider than sternite eight and its apex is truncate in *A. juni*.

*Amamiclytus wuxingensis* sp. nov. can be distinguished from *A. mimicus* by the absence of the white band on the pronotal base and the presence of a white band on the elytral apex. On the other hand, *A. mimicus* has a white band at the pronotal base but no white band on the elytral apex. The white spot behind the suture is short and the white band before the middle of elytra is distinctly arcuate in *A. wuxingensis* sp. nov., while they are long and obliquely transverse, respectively, in *A. mimicus*.

#### Description.

**Figure 1.**
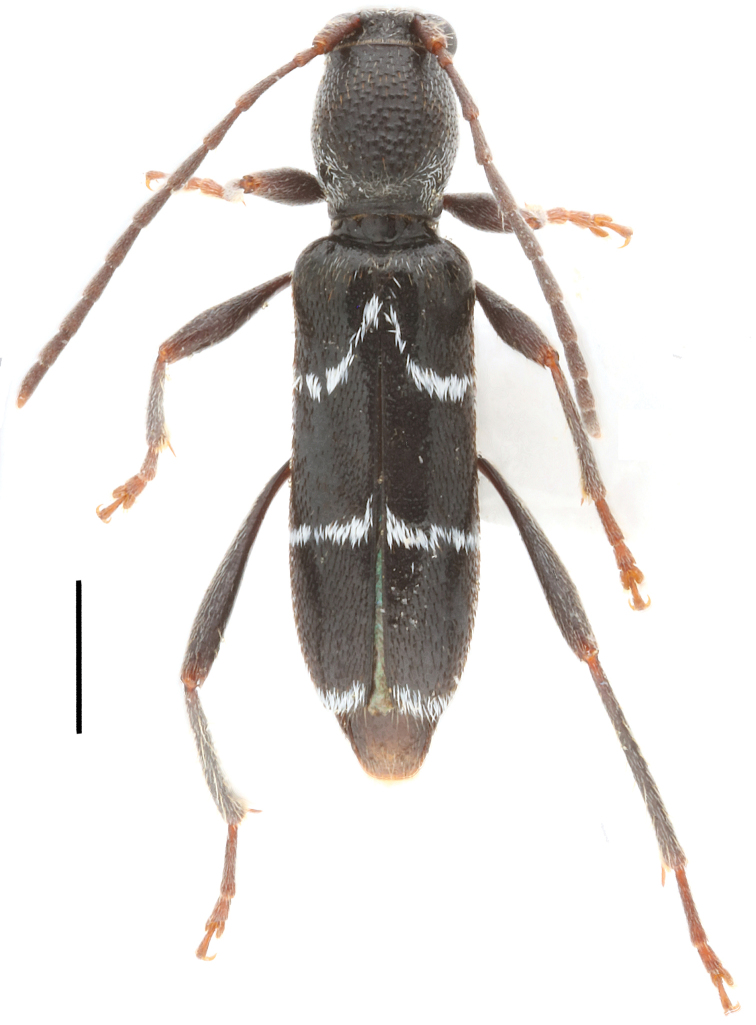
Habitus of *Amamiclytus wuxingensis* sp. nov., holotype (dorsal). Scale bar: 1 mm.

**Figure 2.**
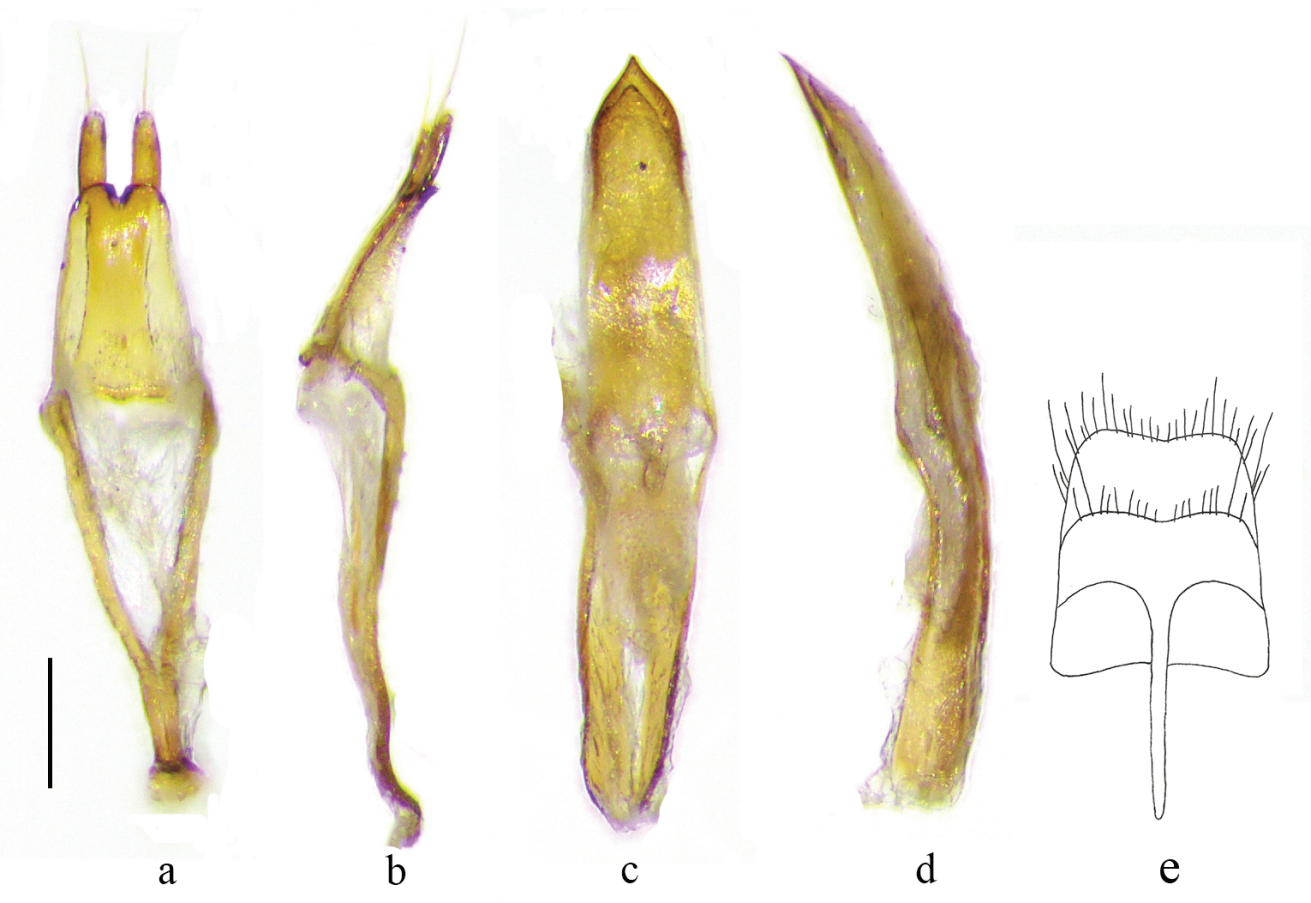
Male genitalia of *Amamiclytus wuxingensis* sp. nov. **a, b** tegmen **c, d** median lobe **e** line drawing of abdominal segment eight (**a, c, e** ventral view **b, d** lateral view). Scale bar: 0.2 mm.

**Male. *Body*** (Fig. 1), length: 3.5–5.0 mm (*N* = 3). Black, glossy in general, dark brown in mouthparts, antenna, abdomen and legs. Body sparsely clothed with long pale hairs, which are darker on disc of pronotum. *Maculations*: Pb absent though sparsely covered with white hairs on basal third of pronotum; B absent; S on basal 1/8, longitudinally, short; La on basal 2/5 of elytron, obliquely arcuate and reaching S; Lp on apical third of elytron, transverse, slightly oblique, complete and reaching suture; A distinct, slightly wider than Lp; Msl distinct; Mss sparse; Mta distinct, dense on apical third of metepisternum and along posterior margin of metasternum and on hind coxae; V1 distinct, dense on sides of posterior margin of ventrite; V2 nearly absent, only sparsely with white hairs; V3 and V4 absent. ***Head***: Frons nearly square, longer than wide, narrowest at the middle of lower eye lobes, clothed with sparse and long hairs with a pattern extending outwards from center of frons; clypeus slightly convex; vertex gradually raised towards antennal insertions; occiput convex with dense large punctures and clothed with sparse short pale hairs; antennae thin and relatively short, reaching 2/5 of elytral apex, clothed with long pale hairs on scape and mostly short but denser pale hairs on other antennomeres, long spinous hairs at internal apices of antennomeres 3, 4 and 5; antennomere 3 distinctly longer than scape and antennomere 4. ***Thorax***: Pronotum slightly longer than wide, constricted abruptly at base; disc slightly raised and convex, matted with sparse granules; scutellum long triangular, slightly acute at apex; prosternum with sparse white hairs covering most of prosternum except the frontal margin of prosternum and the prosternal process. ***Elytra***: nearly parallel-sided, rounded at humeri, obliquely truncated at apex; disc almost evenly convex except depressed parts at basal fifth near humeri; sparsely fine punctured. ***Legs*** relatively short and slender, with hind femora gradually swollen apically, exceeding elytral apices at apical sixth. ***Abdomen*** sparsely fine punctured, with sparse long pale hairs. ***Male genitalia*** (Fig. 2): Median lobe slightly arcuate in lateral view. Dorsal plate slightly narrower than ventral plate in apical fourth, narrowed down to a point at apex. Ventral plate also narrowed to a sharp point at apex. Median struts about half the length of median lobe, widest at base, constricted to basal fifth, nearly parallel for remainder. Tegmen elongate, slightly shorter than median lobe; parameres about 2/5 the length of tegmen, each paramere nearly parallel-sided, slightly narrowed in external side in apical fifth, rounded at apex, provided with numerous short setae and a few relatively long setae at apex; basal ridge slightly raised. Tergite eight elongate and quadrate, slightly narrowed towards apex, which is rounded at sides and concave at the middle, with short to long sparse setae. Sternite eight as wide as tergite eight, concave at the middle as tergite eight, apical margin bi-arcuately rounded, and provided with sparse short to long setae.

#### Etymology.

The name of the new species, *wuxingensis*, refers to the type locality, Wuxing Village, Guizhou Province, China.

#### Note.

Niisato and Han (2013) discussed the wide gap in the distribution of *Amamiclytus* in South China and Southwest China and they expected to discover further members of *Amamiclytus* in these areas. The locality of *A. wuxingensis* sp. nov. falls in this wide distribution gap. Description of *A. wuxingensis* confirms and continues the expectation of discovering more members of *Amamiclytus* in these areas.

##### Key to species of the genus *Amamiclytus* from East Asia

**Table:** 

1	Pronotum longer than wide, weakly or moderately arcuate at sides, usually provided with a white basal band	**2**
–	Pronotum as long as wide, usually strongly arcuate at sides, without white pubescence along basal margin	**7**
2	Elytra glossy	**3**
–	Elytra matted	**6**
3	Elytra provided with white spot near suture behind scutellum	**4**
–	Elytra without white spot near suture behind scutellum	**5**
4	Elytra strongly glossy, white band on base of pronotum distinct, white band on mesosternal process almost absent	***A. nobuoi* Niisato & Han, 2011**
–	Elytra slightly glossy, white band on base of pronotum not distinct, white pubescence on mesosternal process distinct	***A. wenshuani* Niisato & Han, 2013**
5	Elytra moderately glossy, without long pale hairs; frons distinctly longer than wide; erect pale hairs on hind tibiae sparse and not so long	***A. subnitidus* Niisato & Han, 2011**
–	Elytra strongly glossy, scattered with a few erect long pale hairs; frons almost as long as wide; erect pale hairs on hind tibiae dense and long	***A. setiger* Niisato & Han, 2011**
6	Pronotum provided with a dense basal white band; elytra without white spots near suture behind scutellum	***A. nubilus* Niisato & Han, 2011**
–	Pronotum only sparsely clothed with white pubescence near basal margin; elytra with a white spot near suture behind scutellum	***A. hirtipes* (Matsushita, 1940)**
7	Ante-median white bands on elytra usually reaching white spot behind suture	**8**
–	Ante-median white bands on elytra not reaching white spot behind suture	**9**
8	Ante-median white bands on elytra reaching white spot behind suture in a rather oblique way from middle of elytron; post-median white bands on elytra arcuate; prosternal process only with sparse hairs; mesosternal process without white hairs	***A. juni* Niisato & Han, 2011**
–	Ante-median white bands on elytra strongly arcuate and reaching white spot behind suture; post-median white bands on elytra transverse, slightly oblique; prosternal process without white hairs; mesosternal process with sparse white hairs	***Amamiclytus wuxingensis* sp. nov.**
9	Abdomen without lateral white bands on ventrites 3 and 4	***A. limaticollis* (Gressitt, 1939)**
–	Abdomen with lateral white bands on ventrites 3 and 4	***A. yulongi* Niisato & Han, 2011**

## Supplementary Material

XML Treatment for Amamiclytus wuxingensis
